# Don’t mind the gap: how non-naturalists should explain normative facts

**DOI:** 10.1007/s11098-025-02312-0

**Published:** 2025-05-05

**Authors:** Singa Behrens

**Affiliations:** Department of Philosophy, Bielefeld University, Bielefeld, Germany

**Keywords:** Non-naturalism, Grounding, Normative facts, Reasons-First approach

## Abstract

In this paper, I present and defend a novel way for non-naturalists to account for the sui generis status of normative facts, which is consistent with the claim that contingent normative facts obtain in virtue of non-normative facts. According to what I call unsupplemented partial ground approach, non-derivative normative facts have non-normative partial grounds, but are not fully grounded in any collection of facts. This view entails that an explanatory gap separates the normative from the non-normative domain. I argue that this account provides non-naturalists with a metaphysically coherent response to the challenge of accounting for explanatory dependence relations between two domains while positing metaphysical discontinuity (explanatory challenge), and avoids serious objections that alternative non-naturalist accounts face. Moreover, I show that the unsupplemented partial ground approach is an attractive option for the popular Reasons-First approach, which is often, but I argue prematurely, considered a particularly promising account for non-naturalists.

## Introduction

Normative non-naturalists face a well-known, but intricate problem: the *supervenience challenge*. According to non-naturalism, normative facts are of their own kind—or as it is often put *sui generis*.^1^ This claim seems to be in tension with what is often treated as a meta-normative datum: There cannot be changes to the normative without changes to the non-normative (*normative supervenience*). The challenge for non-naturalists is to explain how facts of radically different kinds can be modally so closely related. Two main strategies have been pursued in the literature to meet this challenge: Either non-naturalists have provided a metaphysical explanation of the modal relation in question, or they have denied the original supervenience claim, for example by weakening its modal strength. There is, however, a kindred, but separate challenge to non-naturalism that is equally pressing and that is the focus of this paper: *the explanatory challenge*.

The sort of sui generis status that non-naturalists have in mind excludes that the normative domain and its complement, the non-normative domain, are metaphysically continuous.^2^ Metaphysical discontinuity, however, seems to exclude an appealing and wide-spread claim that is present in almost all theories in normative ethics: Things possess their normative properties *in virtue of* their non-normative properties.^3^ That is, at some point, all normative explanations bottom out in the non-normative domain. So, irrespectively of how non-naturalists deal with the supervenience challenge, they owe us an explanation of how the sui generis status of normative facts squares with the intuitively highly plausible claim that normative facts obtain in virtue of non-normative facts. This paper defends a novel solution to the explanatory challenge to non-naturalism.

Non-naturalists have argued that their view does not generally exclude explanatory links between the normative and the non-normative, but instead calls for some sort of explanatory gap.^4^ The difficulty is to provide a coherent metaphysical story about this gap. On the account that I am going to propose in this paper, the explanatory gap is an unbridgeable gap. In other words, all normative facts are *partially* grounded in non-normative facts, but normative facts that are non-derivative within the normative domain are *merely* partially grounded. That is, they have partial grounds, but are not fully grounded in any collection of facts. The *Unsupplemented Partial Ground Approach* (‘UPGAp’ for short) accommodates the core non-naturalist commitments, but avoids pressing objections against alternative attempts to deal with the explanatory challenge.^5^

It is far from trivial for non-naturalists to specify exactly what role non-normative facts play in explaining normative facts. Two prominent approaches in the metaethical debate are *bridge-law non-naturalism* and the *normative-ground approach*.^6^ According to bridge-law non-naturalism, non-normative facts *partially* metaphysically ground normative facts, while normative laws supplement non-normative partial grounds thereby bridging the explanatory gap. According to the normative-ground approach, non-normative facts *fully* *normatively*, rather than metaphysically, ground normative facts. Both views face serious objections that I briefly outline in Sect. 2. While the views differ in important aspects, they are based on a common assumption: Non-naturalists must provide an account of what *fully* grounds normative facts. I am going to argue that non-naturalists should reject this assumption. This move requires a modification of the orthodox notion of partial ground according to which a partial ground is always part of a full ground.^7^ Such a refined notion of partial ground is vividly discussed in the recent grounding literature (Leuenberger 2020; Trogdon and Witmer 2021). I introduce that notion in Sect. 3. In Sect. 4, I show how non-naturalists can account for the sui generis status of normative facts in terms of unsupplemented partial ground in a way that gets them all they are committed to, while avoiding key objections to alternative approaches. I briefly discuss the account’s metaethical consequences in Sect. 5. Finally, in Sect. 6, I explain why UPGAp is of particular interest to a non-naturalist Reasons-First approach. This result is noteworthy because Reasons-Firsters are often taken to be in a better position than non-naturalist alternatives to respond to the explanatory challenge.^8^

## Normative grounds and normative grounding

Non-naturalist accounts face several objections. Some objections target non-naturalism more generally, such as the explanatory challenge; others are more specific to particular non-naturalist accounts, such as objections that target the role of laws in law-based non-naturalism. In this section, I consider two influential non-naturalist accounts and the problems they face. This will allow me to contrast the *Unsupplemented Partial Ground Approach* with these accounts, and to show that it avoids the objections they face altogether.^9^ However, the problems discussed in this section deserve a deeper discussion than I can provide in this paper. What I hope to do, though, is to show that non-naturalist accounts often incur fairly general metaphysical commitments that go beyond the non-naturalistic core in order to respond to the explanatory challenge, e.g., by adopting a particular view of the grounding role of laws. By contrast, the view that I’m going to propose—I shall argue—stays close to the core of non-naturalism. In what follows, I first consider bridge-law non-naturalism and briefly outline an objection raised by Berker (2019), according to which laws cannot bridge the explanatory gap. Second, I consider the normative-ground approach and then build on a critique by Rosen (2017c), according to which this view entails implausible grounding hierarchies. Finally, I will briefly explain why non-naturalist approaches that try to solve the explanatory challenge by arguing that non-naturalism is compatible with the claim that contingent normative facts are *fully* grounded in non-normative facts are in danger of granting too much to naturalists.

According to bridge-law non-naturalism, non-normative facts *partially* metaphysically ground normative facts, while a full ground of a normative fact includes normative bridge-laws that supplement the non-normative partial grounds. Consequently, normative laws bridge the explanatory gap between the normative and the non-normative domain.

- **Bridge-law non-naturalism**: Particular normative facts are fully grounded in the combination of (i) general normative principles and (ii) various particular facts about the instantiation of the relevant non-normative properties or relations.^10^

Bridge-law non-naturalism has appealing features. It can do justice to practice in normative theorizing, and on the face of it, it seems to align well with explanations of normative judgements in everyday thought and talk. For example, that Sarah ought to help her sister is explained by—let’s suppose—the non-normative fact that Sarah promised to help her sister and the bridge-law that people ought to do what they have promised to do. Moreover, it has been argued that the modal status of normative laws explains why the normative supervenes on the non-normative.^11^ That said, the view faces important objections. Berker (2019) argues that bridge-law non-naturalists face a dilemma. Berker’s objection in a nutshell is this: Normative laws are supposed to close an explanatory gap, but they are either inapt to close the gap, or they close it by stating that there is no gap to be closed. In other words, either there is no work (of the relevant kind) or normative laws fail to do it. Let me explain the objection in more detail.

Bridge-law non-naturalism entails that normative laws are part of the pertinent explanans, but this leaves it open whether they are also *explanatory in content*, i.e., containing explanatory notions such as ‘because’.^12^ Berker argues that both options are problematic.

On the first horn of the dilemma, we assume that normative laws do not contain explanatory notions. Here is one of Berker’s examples: Necessarily, an action is required *if and only if* the action maximizes happiness.^13^ According to Berker, this view entails implausible grounding claims. He correctly stresses that, in general, facts about properties of entities are not grounded in necessitation principles together with facts about the instantiation of sufficient conditions. To illustrate this point, Berker gives the following example: The fact [The ball *B* is red and round] together with the principle [Necessarily, if the ball is red and round, the ball is red] do not ground the fact [*B* is red].^14^ One might worry that Berker’s example is disanalogous to the normative case. In contrast to the normative case, [*B* is red and round] does not even partially ground [*B* is red]. However, we can easily give examples that possess the relevant structure. Given common assumptions about grounding conjunctive facts, facts of the form [$P\, \& \,Q$] are partially grounded in each of the conjuncts. Accordingly, [*B* is red] partially grounds [*B* is red & (grass is green or it is not the case that grass is green)]. Moreover, the following principle is true: Necessarily, *B* is red if and only if (*B* is red, and grass is green or it is not the case that grass is green). In this case, we have a partial ground and a corresponding necessitation principle, but still the corresponding full-grounding claim is implausible. As Berker puts it: ‘necessitation is the wrong sort of relation to underwrite explanation’.^15^

On the second horn, we assume that normative laws contain explanatory notions. Here again is Berker’s example: Necessarily, an action is required if, only if, and *because* the action maximizes happiness.^16^ According to Berker, laws that are explanatory in content can only make redundant contributions to explanations of normative facts. To see why, consider the following example: [Action *A* is required] is grounded in [*A* maximizes happiness] together with [Necessarily, an action is required if, only if, and because the action maximizes happiness]. Since grounding is assumed to be factive, the grounds actually obtain. Consequently, *A* maximizes happiness and the normative law is true. This in turn entails that *A* is required *because* it maximizes happiness. Berker assumes that normative laws that contain explanatory notions specify relations of *full* ground. Consequently, *A* is required *fully* because *A* maximizes happiness. This is a full non-normative ground. Thus, the normative law’s distinctive contribution is a redundant one: It need not be added to non-normative facts that already provide a full ground (though, we may add it).^17^ If Berker is correct, normative laws can only play a redundant grounding role.

Note that, to reach that conclusion, we need not assume that bridge-laws specify relations of full ground. Suppose normative laws specify relations of *partial* ground. It is still implausible that normative laws bridge an explanatory gap between non-normative partial grounds and normative groundee. Consider the general grounding principle (CON) according to which necessarily, an entity *x* is *F* and *G* only if and *partially because* *x* is *F*. Given our previous assumptions, [*B* is red] partially grounds [*B* is red and round]. However, adding (CON) to [*B* is red] does not bring us any closer towards a full ground of [*B* is red and round]. To be clear, we might think that such a general principle is needed to *back* grounding relations, but adding (CON) does not add anything in terms of the completeness of the ground. Berker’s arguments support the general claim that to think of laws as part of a complete ground of a particular fact amounts to a confusion of levels.^18^

A non-naturalist alternative that is based on a distinctively *normative* grounding relation *guided* by normative laws is defended in Bader (2017).

- **Normative-ground approach**: Any particular normative fact is fully normatively, but not metaphysically grounded in non-normative facts.^19^

On that approach, there are at least two distinct grounding relations: metaphysical and normative grounding. A number of questions concerning the individuation of grounding relations arise in this context.^20^ For our purposes, these questions may be set aside. Instead, I shall focus on an objection to the normative-ground approach that has been put forward by Rosen (2017c).

The normative-ground approach entails that some normative facts, unlike other facts about complex entities, are not metaphysically grounded from below. The approach entails that normative facts like [Sam ought to keep her promise] are metaphysically on a par with candidates for fundamental facts such as facts about atoms and charges.^21^ In other words, from a metaphysical point of view, nothing explains why Sam has the obligation in question. I agree with Rosen that this result is hard to swallow. Moreover, there is an additional aspect of this criticism. It is a live possibility that there is no metaphysically fundamental level of reality, for example, because facts about wholes are grounded in facts about their parts and the world is gunky, i.e., there are no atomic parts. Yet, the normative-ground approach excludes this possibility. It entails that there is a fundamental level because there are normative facts that are metaphysically ungrounded. Note that the objection presupposes a link between fundamentality and metaphysical ground that might be rejected. Instead, one might argue that a fact *F* is fundamental iff for all *i*, *F* is not $grounded_{i}$ where we quantify over all flavors of ground. On that approach, facts about charges might turn out to be fundamental, but facts about obligations do not. It is not clear, however, that this response really helps non-naturalists. The normative-ground approach entails that particular normative facts are *metaphysically* fundamental and *metaphysically* on a par with facts about charges. *This* objection has still significant force.

Let me briefly discuss a natural response strategy. Rosen (2017c) suggests a combination of the two approaches: We can be bridge-law non-naturalists while assuming that normative laws are stated in terms of normative grounding. Applied to our previous example, this would look like this: [Sam ought to help] is metaphysically grounded in [Sam promised to help] together with [As a matter of normative necessity, the fact that *x* is a promise normatively grounds the fact that *x* is obligatory]. While I cannot do full justice to this account here, I agree with the concerns discussed in the literature concerning questions about the interaction between distinct grounding relations.^22^ Moreover, I want to briefly raise another concern that is slightly different in focus: The view may not give non-naturalists the kind of discontinuity that they need.

Recall that the sui generis status of the normative is threatened by an immanent continuity with the non-normative domain. A concern about the original bridge-law account is that the normative laws stated in terms of metaphysical ground are redundant, which means that the view entails metaphysical continuity between non-normative and normative facts. A similar concern can be raised against laws stated in terms of normative ground (hereafter ‘n-ground’). The proposal may avoid redundancy, but cannot exclude continuity. Let me explain: First, note that the laws specify *full* n-grounds.^23^ This does not give us *metaphysical* continuity, but it entails that normative and non-normative facts are continuous in a different sense that corresponds to the relevant modality. Second, in light of Rosen’s own reason to reject the normative-ground approach, it is plausible that for every metaphysically possible world and normative fact *N* we have a law that specifies *N*’s full n-ground. In other words, for every metaphysically possible world and normative fact we have some non-normative facts that are a complete explanation base and thus (in some sense) continuous with it. How does this result fit with the claim that normative facts and the *class* of non-normative facts are metaphysically discontinuous?

Let me briefly illustrate the concern with an example: Suppose there is *natural grounding* (hereafter ‘nat-grounding’), which, for the present purposes, can be thought of as corresponding to the kind of modality associated with the laws of nature. Suppose also that pain facts are *fully* nat-grounded in facts about C-fibre firings (*C*-facts) in our world. For simplicity, we assume that there are only two other types of metaphysically possible worlds: i) worlds in which pain facts are *fully* nat-grounded in neurophysiological state *A* and ii) worlds in which pain facts are *fully* nat-grounded in neurophysiological state *B*,^24^ Importantly, *A*-facts, *B*-facts, and *C*-facts are all neurophysiological facts or more broadly physical facts. Now, in every metaphysically possible world, pain facts are fully explained in terms of physical facts. Given this scenario, it would be surprising to claim that pain facts and physical facts are metaphysically discontinuous—at least, we would like to hear a reason why.^25^ After all, the physical domain always provides a complete explanation base. Since there seems to be no reason to treat the normative case differently, it is not clear that the account satisfactorily meets the explanatory challenge.

Finally, let me return to non-naturalist accounts, according to which the claim that contingent normative facts are fully metaphysically grounded in purely non-normative facts is compatible with non-naturalism.^26^ Call this form of non-naturalism *compatibilism*. While the details of the approaches differ, compatibilists argue that non-naturalism is best understood as the claim that the essence of some normative properties ineliminably involves a normative component.^27^ According to the authors, this claim is compatible with contingent normative facts being fully metaphysically grounded in purely non-normative facts. Accordingly, compatibilists claim that (a) contingent normative facts have full non-normative grounds, but (b) are not metaphysically continuous with non-normative facts because their nature or essence is something over and above their non-normative ground.

Compatibilism has been criticized for granting too much to their opponents, especially to non-reductive naturalists.^28^ Reductive and non-reductive naturalists disagree precisely on whether the nature of the normative is exhausted by the non-normative such that reduction is warranted. While this is not the place to present a comprehensive argument against compatibilism, an example might help to see the concern: Consider disjunctive facts such as [Tim is a beaver or a rhino]. Note that nobody is non-naturalist with respect to disjunctive facts. [Tim is a beaver or a rhino] seems to be perfectly metaphysically continuous with [Tim is a beaver] and [Tim is a rhino]. However, as far as disjunctive facts are concerned, it is common to accept claims analogous to (a) and (b) as made by compatibilists. First, it is common to assume that disjunctive facts are fully metaphysically grounded in their true disjuncts. So, [Tim is a beaver or a rhino] is fully metaphysically grounded in the non-disjunctive fact that—let’s say—Tim is a rhino ((a)). Moreover, it is hard to deny that it is part of the essence of disjunctive facts to involve disjunctions. That is what it is to be a disjunctive fact as apposed to a non-disjunctive fact. Thus, the nature or essence of disjunctive facts is something over and above their full ground, in our case [Tim is a rhino] ((b)). But if claims like (a) and (b) do not license non-naturalism about disjunctive facts, why should they license normative non-naturalism? Of course, this is no conclusive argument, but it motivates an alternative solution to the explanatory challenge. In sum, the discussed non-naturalist approaches face serious objections that are based on general ground-theoretic considerations.

## Unsupplemented partial ground

The non-naturalist accounts are based on the assumption that non-naturalists must specify full grounds of normative facts. My aim is to argue that non-naturalists should reject this requirement. Instead, they should take the claim that there is an explanatory gap between the two domains at face value. Any particular normative fact that is non-derivative within the normative domain has non-normative partial grounds, but is not fully grounded in any collection of facts. This view requires a notion of partial ground that I shall introduce in this section.

Commonly, full ground is taken as primitive. Partial ground is defined in terms of full ground as follows: A fact *G* partially grounds a fact *F* iff for some (possibly empty) collection of facts $\Gamma$: $\Gamma , G$ fully grounds *F*.^29^ That partial grounds are always part of a full ground, however, has recently been questioned in the grounding literature.^30^ In some cases, it is intuitively plausible to say that some fact helps to explain another fact, while the fact does not cover all the explanatory work, and at the same time, it is unclear what we could add to the fact in order to obtain a full ground. The authors suggest that in those cases, we face instances of *unsupplemented partial ground*. To better grasp the idea, let us consider some of the examples that are discussed in the literature. Note that I do not commit myself to the claim that the examples are indeed cases of unsupplemented partial ground. However, I take them to motivate a definition of partial ground that is compatible with such cases.

Totality facts provide one kind of example.^31^ Consider the fact [Ann, Bob, and Chan are all the things that exist]. The fact [Ann exists] plausibly helps to explain why Ann, Bob, and Chan are all the things that exist. The same holds for [Bob exists] and [Chan exists]. But do these facts together provide a full ground of the totality fact in question? If we think that grounding necessitarianism is true, i.e., that full grounds necessitate what they ground, then plausibly not. To see why, consider a possible world where something else, for example, Dora, exists. That Dora exists is compatible with the fact that Ann, Bob, and Chan exist. It is thus compatible with the grounds. However, it is incompatible with the totality fact [Ann, Bob, and Chan are all the things that exist]. Note that we need not incur any commitments to grounding necessitarianism to argue for the incompleteness of the grounds. Part of what has been said when we claim that Ann, Bob, and Chan are all the things that exist is that nothing besides Ann, Bob, and Chan exists. That, it seems, is not explained by facts about Ann’s, Bob’s, and Chan’s existence. If this reasoning is correct, the three facts together only provide a partial ground. However, there does not seem to be any fact that could supplement the partial grounds that is not already the totality fact itself.

Another kind of example is based on fundamental properties. Let us suppose that having spin-down is a fundamental property that atoms can, but need not possess. They might have some other incompatible property, e.g., having spin-up, instead. If we consider the fact that atom *A* has spin-down, it seems plausible that the fact that *A* exists helps to explain why A has spin-down. However, this fact is plausibly no full ground of the fact that *A* has spin-down. After all, *A* could have had spin-up instead. Moreover, like before, it is unclear what could be added to the existence fact to explain why *A* has spin-down that is not already the grounded fact itself.

One way to make room for the intuitive assessment of the examples is to take the notion of partial ground to be prior to the notion of full ground.^32^ Another way to go is to assume a notion of full ground as primitive, but allow for variation in the notion of full ground.^33^ A number of ground-theoretic questions arise in this context, but fortunately, we can sidestep these issues. For our purposes, it suffices to assume that there can be partial grounds that cannot be supplemented by other facts so that we obtain a full ground in the strict sense. If facts are unsupplemented partial grounds in this sense, I shall also say that they *merely* partially ground some other fact.

Before we return to the normative domain, let me emphasize once more that, so far, we are not committed to the claim that there actually are any instances of unsupplemented partial ground. The refined notion of partial ground merely makes room for the possibility that some facts make an explanatory contribution without being part of a complete ground. In the next section, I shall apply the notion of unsupplemented partial ground to the normative domain and investigate the prospects of such an account.

## The normative domain on partial ground

In this section, I outline an account according to which any particular normative fact that is non-derivative within the normative domain is merely partially grounded in non-normative facts. Before, it is important to clarify the aims of the outlined approach. Given that non-naturalists posit a sui generis kind of fact, it is not surprising that non-naturalist accounts incur some additional commitments. So, I do not set out to provide an account that avoids any such commitments. There is no free lunch for non-naturalists. However, we already saw that extant non-naturalist accounts face serious objection based on *metaphysical*, or more specifically, *ground-theoretic* considerations. If the criticism is correct, bridge-law non-naturalism confuses the roles played by grounds and played by laws. On the other hand, the normative-ground approach entails metaphysically implausible results, namely implausible grounding hierarchies. My aim is to show that the account that I am going to propose provides non-naturalists with a metaphysically coherent response to the explanatory challenge that takes into account all non-naturalistic core commitments, but that avoids the outlined ground-theoretic objections.

Let me first state the *Unsupplemented Partial Ground Approach*, before I discuss its two components.

- **UPGAp**: (1) Any particular normative fact is partially, yet not fully grounded in non-normative facts. (2) Any particular normative fact that is non-derivative within the normative domain has non-normative partial grounds, but is not fully grounded in any collections of facts.

According to UPGAp’s first part, non-normative facts do not provide *complete* grounds of particular normative facts. This claim accounts for the non-naturalist’s core commitment that the two domains are metaphysically discontinuous. Moreover, it grants that all particular normative facts are *partially* grounded in non-normative facts. Thus, it accounts for the common assumption that particular normative facts are not ungrounded. UPGAp’s original claim is its second part. Some normative facts are not fully ground in *any* collection of facts, though they are partially grounded. That is why normative facts are sui generis. Let me explain why the second part is restricted to facts that are non-derivative within the normative domain. Suppose the normative fact [Sarah ought to help her sister] is *fully* grounded in the normative fact [Sarah has decisive reason to do so]. If UPGAp would be incompatible with grounding-claims of that kind, it would be a non-starter. Thus, UPGAp cannot state that *all* particular normative facts lack complete grounds. However, facts that are non-derivative within the normative domain do not have any complete *normative* grounds by definition. UPGAp’s first part entails that they do not have any complete *non-normative* grounds either. So, UPGAp’s distinctive claim is that non-derivative normative facts, despite lacking any complete grounds, do have partial grounds. Suppose [Sarah’s act is good] is non-derivative within the normative domain. According to UPGAp, some non-normative facts partially explain why Sarah’s act is good, for example, that Sarah’s act prevents harm. However, UPGAp entails that this fact does not fully explain why Sarah’s act is good. Indeed, it entails that nothing fully explains it. The normative fact is genuinely new compared to the non-normative domain, but not entirely detached from it. This entails an unbridgeable explanatory gap between the two domains which is at the heart of non-naturalism.

One might object that even if we grant that there are examples of merely partial ground, it just doesn’t seem like normative facts are among these types of facts.^34^ The explanation ‘Sarah ought to help because she promised to do so’ does not seem incomplete in the way that ‘Atom *a* has spin-up because it exists’ may seem incomplete.^35^ So one might wonder whether non-naturalists have a positive reason for thinking that normative facts are merely partially grounded, i.e., that (i) the relevant ground is only partial rather than full, and (ii) there cannot be another partial ground such that together they fully ground the relevant fact. In order to see that non-naturalists do have such a positive reason, note that non-naturalists often motivate their view by what has come to be known as the *just-too-different intuition*, according to which normative and non-normative properties are fundamentally different.^36^ The normative is set apart from all non-normative properties or relations by what we might call their “action-guiding” character, or their relationship to reasons, etc. Some part or aspect of the normative is not covered by non-normative facts. In other words, the normative is *something over and above* the non-normative. So, insofar as aspects of the normative explanandum or groundee are not covered, any grounding explanation in non-normative terms seems to be incomplete.^37^ Thus, the non-naturalist’s commitments give non-naturalists a positive reason for thinking that no *purely non-normative* grounding explanation of normative facts is complete (i).

Second, general considerations about laws, according to which laws are outside of grounding hierarchies, give non-naturalists positive reason for thinking that laws are incapable of completing incomplete grounding explanations. Finally, since we are considering normative facts that are non-derivative in the normative domain, no contingent normative fact could complete the grounding explanation. Thus, non-naturalists have positive reason to think that there cannot be any other partial ground that together with the non-normative facts fully ground the relevant normative fact (ii). The following example (to which I will return later in more detail) *prima facie* parallels the atom example, thus supporting their similarity and nicely illustrating the positive reasons just described: Consider the normative fact [*p* is a reason for *S* to $\phi$] and suppose this fact is a fundamental normative fact. It seems plausible that this fact obtains in part because *p* obtains. However, there seems to be no other fact that could team up with *p* to fully explain the reasons fact (just like the atoms existence partially explains the spin fact, but since spin is a fundamental property nothing could be added such that we obtain a full ground). What we might call the “action-guiding” character of normative facts is, I think, a positive reason for non-naturalists to hold that some normative facts are cases of merely partial ground.^38^

At this stage, one might be dissatisfied with the account’s consequence that there are incomplete explanations. In one sense, UPGAp entails that this is how the world is. However, it is independently plausible that some partial explanations are better than others. So, even in cases of unsupplemented partial ground, we might regain distinctions that are close to our talk about partial and complete grounds by appealing to parts of a ‘fuller’ ground. Suppose Sarah’s act is good partially because she helps her sister and partially because her sister is in distress. A grounding claim that merely cites the fact [Sarah’s sister is in distress] is less exhaustive than the previously outlined grounding claim that cites both partial grounds. These considerations motivate a notion of an exhaustive ground. A partial ground is an *exhaustive* ground if it is not part of a fuller ground. Does this mean that the notion of an exhaustive ground collapses into the notion of a maximal ground? Cases of overdetermination show that it does not because full grounds which intuitively should come out as exhaustive grounds, are not maximal in those cases.^39^ A satisfactory account of exhaustive ground is an intricate matter. However, the notion of a maximal partial ground—even if not fully satisfying as a general account—would serve present purposes. This is why I postpone a detailed discussion of exhaustive grounds to another occasion and instead rely on an intuitive grip on the distinction.

Proponents of UPGAp might employ the notion of an exhaustive ground to regain a notion of complete grounding explanations: Complete grounding explanations cite exhaustive grounds. This proposal aligns well with one of Berker’s comments when he discusses whether moral laws specify relations of full or partial ground. While Berker assumes the orthodox notion of ground and thus must be read as referring to full grounds, UPGAp is compatible with the following passage:

[...] statements that pick out a single partial ground for a given sort of particular moral fact but fail to specify any of the other partial grounds that team up with that one partial ground don’t qualify as statements of moral law. Instead what we are looking for is a specification of *all the partial grounds*.^40^

How does UPGAp respond to the objections raised against alternative non-naturalist accounts? First, it should be clear that UPGAp resists the objection that non-naturalists grant too much to naturalists by granting that non-normative facts somehow fully ground normative facts. Second, UPGAp is compatible with the widespread metaphysical view that laws *back or guide* grounding relations rather than being part of the ground. Third, UPGAp is not committed to grounding pluralism. Forth, it entails plausible grounding hierarchies to which we now turn in more detail. According to one of the objections, it is implausible that facts like [Sarah ought to help] are metaphysically ungrounded and thus belong to the metaphysically most fundamental level of reality. To see that UPGAP does not entail these implausible consequences, it will be useful to consider a distinction between strongly and weakly fundamental facts made in Leuenberger (2020). Strongly fundamental facts do not have any grounds, while weakly fundamental facts may have partial, but do not have any full grounds.^41^ UPGAp entails that particular normative facts are at most weakly fundamental. Consequently, facts about atoms might turn out to be strongly fundamental, but normative facts are not. Still, they are not completely derivative; some of them are weakly fundamental. On that account, normative facts are moved up in the metaphysical grounding hierarchy. They are located on higher levels than facts about atoms, molecules, and the existence of agents or actions. The picture captures the informal idea that the normative is something genuinely new compared to the non-normative, but not entirely detached from it.

## UPGAp and the supervenience challenge

While the explanatory challenge to non-naturalism has not received sufficient attention in the literature, the so-called *supervenience challenge* (‘SC’ for short) is vividly discussed. Roughly, normative supervenience is the claim that if two worlds are alike in all non-normative aspects, they are alike in all normative aspects.^42^ The non-naturalist’s commitment to metaphysical discontinuity between the normative and the non-normative domain makes it difficult for non-naturalists to *explain* why the normative bears this *modal* relation to the non-normative domain (if it does). If they assume normative supervenience, non-naturalists seem to be committed to brute necessary connections between discontinuous entities. In this section, I show that UPGAp is compatible with normative supervenience, and in particular, with existing solutions to the supervenience challenge as well as with the denial of normative supervenience. This result illustrates that the explanatory challenge is separate from the supervenience challenge. UPGAp does not go so far as to provide a novel solution to the supervenience challenge, but it is a solution to the explanatory challenge that avoids the problems of alternative accounts. That said, it is important to acknowledge that UPGAp is in no worse position than alternative accounts to meet the supervenience challenge, while it is in a much better position to respond to the explanatory challenge.

It is a natural thought that the situation for UPGAp is even worse than for alternative non-naturalist approaches when it comes to supervenience. In general, partial grounds do not necessitate what they ground, e.g., [*B* is red] is not modally sufficient for [*B* is red and round]. Thus, UPGAp seems to be in a worse position than, say, bridge-law non-naturalism when it comes to SC. One might even think that UPGAp is incompatible with normative supervenience. At this juncture, there are two paths that non-naturalists could take. First, they can reject normative supervenience. Recently, a number of authors have argued against metaphysical supervenience claims.^43^ If one is willing to do so, UPGAp provides a neat basis for arguing and explaining why metaphysical supervenience fails. I take this first path to be a solid position that is supported by independent reasons.^44^ However, UPGAp alone doesn’t settle whether the normative metaphysically supervenes on the non-normative. In what follows, I will argue (i) that UPGAp does not formally rule out normative supervenience, (ii) that in general, it is not implausible for facts in one domain to be merely partially grounded in facts from another domain while supervenience holds, and (iii) that UPGAp can be combined with the same resources that alternative non-naturalist accounts use to meet the *supervenience challenge*.^45^

Let me first show that UPGAp does not formally rule out normative supervenience. Some facts supervene on facts in which they are partially grounded. The examples to follow admittedly have some artificial flavor, but they show that UPGAp is not incompatible with metaphysical supervenience. I take this to be an important intermediate step, and so I must ask for the reader’s patience until we get to the more substantive discussion.

Let *F* be some arbitrary fact, e.g., [*B* is red]. Now, conjoin *F* with a necessarily obtaining fact like [Grass is green or grass is not green]. [*B* is red] partially grounds [*B* is red & (grass is green or grass is not green)], and the latter supervenes on the former.^46^ However, given standard assumptions about grounding conjunctions and disjunctions, the former fact does not fully ground the conjunctive fact, since disjunctions are grounded in their true disjuncts and conjunctions are grounded via both of their conjuncts.^47^ Another kind of example that does not involve necessarily obtaining facts is based on double-negation. Again, let *F* be some arbitrary fact. We assume that *F* fully grounds $\lnot \lnot F$, but not vice versa. Together with the previous assumptions, this entails that $\lnot \lnot F$ merely partially grounds $F\, \& \,\lnot \lnot F$. However, $F\, \& \,\lnot \lnot F$ supervenes on $\lnot \lnot F$. The same structure can be applied (at least in one direction) to all pairwise distinct facts that are necessarily equivalent. For example, [*X* is an equilateral triangle & *X* is an equiangular triangle] supervenes on [*X* is an equilateral triangle], but is only partially grounded in it. The outlined examples, despite their artificial flavor, show that *partial* explanatory success plus modal sufficiency does not guarantee *full* explanatory success. Thus, UPGAp does not formally rule out normative supervenience.

Let me now turn to the question of whether it is plausible to combine UPGAp with normative supervenience. There is an interesting parallel between UPGAp and one of the non-normative candidates for merely partial grounds. As we have already seen, totality facts are a good candidate for merely partially grounded facts: It seems that nothing could be added to [*A*, *B*, and *C* exist] in order to obtain a full ground of [*A*, *B*, and *C* are all the things that exist] that is not already the totality fact itself. The partial grounds of the totality fact are facts about particulars. Note that totality facts also supervene on particular facts. No two possible worlds can differ in what totality facts hold without differing in what particular facts hold. However, no set of particular facts entails a totality fact. Moreover, if totality facts are merely partially grounded, no set of particular facts *fully* grounds a totality fact. Totality facts thus illustrate that, in principle, facts from one domain could be merely partially grounded in facts from another domain, but supervene on the domain to which the partial grounds belong. I suggest that the normative case may be similar. First, note that totality facts and normative facts have been taken to be subject to the same kind of inferential barrier.^48^ We cannot infer a normative conclusion from non-normative premises and likewise we cannot infer a totality conclusions from premises about particulars. Similarly, if normative facts are merely partially grounded in non-normative facts, the normative facts might still supervene on the non-normative domain. An explanatory gap between the domains does not rule out supervenience.

If my arguments so far are correct, then we can combine UPGAp with normative supervenience. Accordingly, UPGAp, together with the assumption of supervenience, faces the *supervenience challenge*. However, non-naturalists can use the same resources that bridge-law non-naturalists or grounding pluralists use to respond to SC, without being subject to the ground-theoretic objections that these approaches face. It is plausible that there is a metaphysical law according to which every totality fact of the form [$a_{1}, a_{2}, \ldots$ are all the things that exist] is (at least) partially grounded in facts of the form [$a_{i}$ exists]. If one holds that metaphysical laws guide or mediate grounding relations, this law guides or mediates all merely partial grounding relations between particular facts and totality facts. Likewise, normative laws may *guide* all merely partial grounding relations between non-normative and normative facts. The only additional assumption non-naturalists need to make in oder to respond to SC is that it is part of the nature of normativity that if it obtains, normative laws obtain which guarantee normative supervenience. However, it is important to note that the laws only specify *partial* grounds, which only give rise to partial grounding relations, which in turn blocks the continuity threat.

Bridge-law non-naturalists and grounding pluralists must assume metaphysically necessary normative laws to respond to SC. Hence, the non-naturalist approaches seem to be on a par with respect to SC. However, UPGAp is generally advantageous because it provides a solution to the explanatory challenge, while avoiding ground-theoretically implausible assumptions (like laws among the grounds). Moreover, UPGAp treats structurally similar cases in a similar way (totality facts). This concludes my discussion of normative supervenience and UPGAp. In the final section, I discuss the application of UPGAp to a particular kind of normative fact, namely normative reasons facts. I take a reasons-based version of UPGAp to be the best version of the view. More importantly, however, the discussion of standard reasons-based non-naturalism will show that the best way to construe this view is along the lines of UPGAp.

## Normative reasons on partial ground

In this section, I turn to the so-called Reasons-First approach, which is of particular interest in the present context because it has been considered a way out for non-naturalists. On the Reasons-First approach, the normative reason relation is a *normative* primitive.^49^ That is, at least some normative reason facts are non-derivative within the normative domain.^50^ Unlike, for example, Ought-First or Value-First approaches according to which ought- or value facts occupy the bottom level of the normative domain, the Reasons-First approach appeals to a normative primitive that does not attribute normative properties to particular acts or agents, but that relates agents and acts to *facts*. The application of UPGAp to normative reasons facts is particularly relevant for at least two reasons: First, it shows that, although some have argued otherwise, appeals to normative reasons alone do not solve the non-naturalist’s problems; second, normative reasons facts are—or so I shall argue—excellent candidates for merely partially grounded facts. In order to explain the appeal of reason-based non-naturalism, let me first make some preliminary remarks about normative reasons.

Normative reasons (henceforth ‘reasons’) support or count in favor of the action or attitude they are reasons for. The canonical form of reasons facts is [[*p*] is a reason for *S* to $\varphi$] where [*p*] is a fact or true proposition (the reason), *S* is a subject, and $\varphi$ is an action or attitude.^51^ To give but one example: [This piece of metal is sharp] is a reason for Sarah not to press her hand against it. On that approach, the reasons relation is factive, i.e., if [*p*] is a reason (for *S* to $\varphi$), it is true that *p*.

It has been argued that the Reasons-First approach provides the resources to avoid objections raised against bridge-law non-naturalism and the normative-ground approach.^52^ While it seems hard to swallow that no feature of Sarah’s act helps to explain why it is valuable (or obligatory), it is intuitively less difficult to make sense of the view that nothing explains why [Sarah promised to help] is a reason for Sarah to help. For example, Berker (2019) writes: ‘But if instantiations of the reason relation can be ungrounded, then those non-naturalists who reject Principles as Partial Grounds [bridge-law non-naturalism in my terminology] can continue to be non-naturalists by taking (at least some) particular facts about reasons to be the *ungrounded* grounders of the normative realm.’^53^ In a footnote, Berker acknowledges that for that view to get off the ground, one has to deny that [[*p*] is a reason for *S* to $\varphi$] is partially grounded in [*p*].^54^ In what follows, I argue that Reasons-Firsters have reasons to accept what Berker wants to deny: Contingent reasons facts, i.e., reasons facts that entail that the reason in question obtains, are partially grounded. If the argument succeeds, Reasons-Firsters who want to be non-naturalists face exactly the problems outlined in the beginning of this paper: Given that reasons facts are partially grounded, they seem to require full grounds, but it is difficult to see what fully grounds reasons facts on a non-naturalist account. So, I will argue that the best way to construe reasons-based non-naturalism is along the lines of UPGAp. I will substantiate this claim by briefly discussing a familiar Reasons-First approach by Scanlon (2014). Since UPGAp allows non-naturalists to avoid the objections, it is a promising approach for Reasons-Firsters.

Let me start with the claim that some reasons facts are (un-)grounded. Why should we think that [[*p*] is a reason for *S* to $\varphi$] is partially grounded in [*p*]? First, let us think of a grounding explanation for another fact, namely [There is a reason for Sarah to $\varphi$]. Here is a promising partial grounding explanation: There is a reason for Sarah to $\varphi$ *because* she promised to $\varphi$. Second, note that the grounded fact is of the form [There is an *F*]. According to a common grounding principle, such existential facts are grounded in their true instances. That is, [There is an *F*] is grounded in facts of the form [*a* is *F*] where *a* is some individual. To get a better grip on this abstract claim, consider the following instance: [There is a female philosopher] is grounded in [Hannah Arendt is a female philosopher]. Back to the argument, the general pattern entails that [There is a reason for Sarah to $\varphi$] is grounded in [[*p*] is a reason for Sarah to $\varphi$]. Third, the grounding principle that I have just mentioned also entails that facts of the form [There is an *F*] are *immediately* grounded in their true instances, while all other facts that ground the existential fact do so *mediately* via grounding one of the instances. If this is correct, [Sarah promised to $\varphi$] mediately grounds the existential fact [There is a reason for Sarah to $\varphi$] via grounding one of its instances. Plausibly, the relevant instance is [[Sarah promised to $\varphi$] is a reason for *S* to $\varphi$]. Hence, [*p*] at least partially grounds [[*p*] is a reason for *S* to $\varphi$].^55^

If the argument is correct, then some contingent reasons facts are partially grounded in non-normative facts. As I have already outlined, this result puts pressure on non-naturalist Reasons-Firsters. What fully grounds contingent reasons facts? A natural candidate for facts that supplement the non-normative grounds of contingent reason facts are non-contingent reason facts. Indeed, according to Scanlon (2014), to say that mixed (i.e., contingent) normative claims are true in virtue of non-normative claims ‘would be misleading insofar as it suggested that they are true only in virtue of the truth of these claims, neglecting the role of pure normative claims in determining how this is the case’.^56^ According to Scanlon’s approach, pure normative claims are not contingent on non-normative facts. In what follows, I consider two construals of Scanlon’s pure normative claims (by Rosen (2017b) and Schroeder (2015)), and argue that both accounts face variants of the objections to bridge-law non-naturalism and the normative-ground approach. I conclude that UPGAp is an attractive position for non-naturalist Reasons-Firsters.

The first construal of Scanlon’s pure normative claims that I shall consider here is discussed in Rosen (2017b). Note that on Scanlon’s account, *R*(*p*, *x*, *c*, *a*) means that *p* is a reason for person *x* in situation *c* to do or hold *a*. Rosen writes:

Start with the contingent normative fact *R*(*p*, *x*, *c*, *a*) and list the non-normative facts upon which it depends for its explanation. These will be facts about the agent, her circumstances, the actions open to her, and so on, all of which we may package as a single non-normative claim $\psi (p, x, a, c)$. The corresponding pure normative principle is then the universally quantified conditional:

- $$\forall p \forall x \forall a \forall c \, \, (\psi (p,x,a,c)\rightarrow R(p, x, a, c))$$

This general truth is not contingent on the non-normative facts, since every relevant condition has been bundled into the antecedent.^57^

Rosen’s proposal is supported by Scanlon’s remark that he takes pure normative claims to be examples of what Fine (2002) calls ‘world-bound normative *conditionals*’.^58^ Based on the outlined analysis, Rosen states that a contingent reasons fact is grounded in the relevant non-normative facts together with pure normative principles.^59^ It is noteworthy that this proposal is bridge-law non-naturalism applied to a particular kind of normative fact, namely a reasons fact. Consequently, this view is subject to the challenges raised by Berker. Moreover, what seemed to be a particular strength of the reasons-based approach, namely that the reasons relation assigns normative significance to non-normative facts, is no longer essential to the approach. On the discussed construal, a *conditional connection* assigns normative significance to non-normative conditions.^60^

The second construal of Scanlon’s pure normative claims that I shall consider is discussed in Schroeder (2015). Schroeder argues that pure normative claims have the form *R*(*p*, *x*, *c*, *a*). Here is one of Schroeder’s examples: Suppose a contingent reason for Sarah to read this book is the fact that Sarah finds this book interesting. According to Schroeder, for that to be true there must be some circumstance *C* such that Sarah is in *C*, Sarah finds this book interesting, and *R* holds of the proposition that Sarah finds this book interesting (*P*), Sarah, *C*, and the action of reading this book (*A*). The pure normative claim is that *R* holds of the stated tuple $\langle P, S, C, A\rangle$.^61^ If non-naturalists use this construal to respond to the explanatory challenge, they encounter a problem that I have discussed in the context of the normative-ground approach. Let me briefly remind the reader of the central concern: Unlike other facts about complex entities, *normative* facts about particular agents and actions are not metaphysically grounded from below given that they are only normatively grounded. On the approach under consideration, *R* holds of particular tuples $\langle p, x, c, a\rangle$. At least some of these claims are not grounded from below. Consequently, at the most fundamental level of reality, we have to appeal to *particular* actions, agents, circumstances that are relevant for action, and complex propositions that are candidates for reasons. Admittedly, any assessment depends on what exactly the ungrounded pure normative truths are. But to illustrate the worry, reconsider the discussed example: *R* holds of the proposition [Sarah finds this book interesting], Sarah, *C*, and the action of reading this book. This is a surprising candidate for a metaphysically fundamental fact.

In sum, Reasons-First approaches do not seem to be in a better position than alternative non-naturalist approaches to meet the explanatory challenge. UPGAp’s central claim is, however, that non-naturalists should reject that normative facts must have full grounds. Contingent reasons facts are partially grounded in non-normative facts, but they are not fully grounded in any collection of facts. This gives non-naturalists what they are committed to, but avoids the ground-theoretic objections that alternative approaches face. At the same time, Reasons-First approaches fit particularly well with UPGAp. It seems as least less difficult to make sense of the view that nothing explains why [Sarah promised to help] is a reason for Sarah to help than to claim that ought- or value-facts are brute. If my arguments are correct, there is something that helps to explain why reasons facts obtain, namely that the reason obtains (possibly in addition to other facts). But nothing fully explains why the non-normative fact is a normative reason. That part remains unexplained; indeed, some might take it to be what constitutes the explanatory gap between the domains. Reasons facts add something genuinely new to the non-normative domain that cannot be fully explained, but they are not explanatorily detached from it.

## Conclusion

Non-naturalists take normative and non-normative facts to be metaphysically discontinuous. Consequently, many non-naturalists deny that normative facts are fully metaphysically grounded in non-normative facts. But if non-normative facts merely partially explain normative facts, what fully explains them? Attempts to respond to what I have called the explanatory challenge by referring to normative laws or to a distinctively normative grounding relation face serious difficulties. I have argued that non-naturalists can avoid these difficulties by employing a notion of unsupplemented partial ground recently invoked in the grounding literature. The view that I have called UPGAp entails that particular normative facts that are non-derivative within the normative domain are partially grounded in non-normative facts, but are not fully grounded in any collection of facts. The view accounts for the non-naturalist’s core commitments, entails satisfying results with respect to the localization of particular normative facts within metaphysical grounding hierarchies, and avoids implausible ground-theoretic consequences. It turned out that UPGAp is of particular interest to the Reasons-First approach because it allows Reasons-Firsters to acknowledge explanatory links between reasons facts and the non-normative facts they depend on without incurring naturalist commitments.

## Data Availability

Not applicable.

## Footnotes

1 This claim is often characterized as a core commitment of non-naturalism, see McPherson (2012: 207). As I am using it here, the term ‘normative’ should be understood broadly to encompass the evaluative and the normative in a stricter sense.

2 McPherson (2012) invoked the term ‘metaphysical discontinuity’ in the context of non-naturalism. What exactly metaphysical discontinuity requires or excludes is controversial. However, some forms of non-naturalism are compatible with the view that normative facts are fully metaphysically grounded in non-normative facts, such as Leary (2021) and Bengson et al. (2024). I will briefly discuss this kind of view and some concerns about its status as genuinely non-naturalist at the end of §2. Part of the aim of this paper is to argue that non-naturalists can coherently deny that normative facts are fully grounded in non-normative facts, which in turn allows for a *robust* form of metaphysical discontinuity.

3 This is what (Väyrynen, 2013) calls ‘the dependence intuition’.

4 See, for example, Rosen (2017a).

5 Note that I do not claim to offer a solution to the supervenience challenge, but as I will argue in §5, UPGAp is on a par with alternative approaches in this respect.

6 Rosen (2017a) argues in favor of bridge-law non-naturalism; Bader (2017) argues in favor of the normative-ground approach.

7 While the account indeed requires a modification of the orthodox notion of partial ground, a variant of that notion can be found in Fine’s (2012, 2017) influential semantics for ground. Most parts of the grounding debate remain unaffected by adopting the refined notion.

8 Thanks to an anonymous reviewer for suggesting that I discuss the explanatory challenge as a separate puzzle.

9 Thanks to an anonymous reviewer for asking me to clarify this.

10 This rather general characterization of bridge-law non-naturalism can be further specified. See, e.g., Rosen (2017a), Berker (2019). For the current purposes, however, the details do not matter.

11 For a careful discussion of the supervenience challenge to non-naturalism, see McPherson (2012). For a recent critical assessment of the law-based strategy, see Bhogal (2024).

12 Following Fogal and Risberg (2020), I call ‘explanatory in content’ what Berker calls ‘explanation-involving’ (Berker 2019: 905).

13 Berker (2019: 908).

14 I shall refer to the fact expressed by the sentence *S* by enclosing it in square brackets.

15 Berker (2019: 912).

16 Berker takes it to be relatively uncontroversial that normative laws are indeed explanatory in content, see Berker (2019: 908).

17 Note that this form of redundancy differs from cases of overdetermination. For example, if the disjunctive fact [$P\vee Q$] is fully grounded in [*P*] and fully grounded in [*Q*], it is fully, but redundantly grounded in [*P*], [*Q*]. Arguably, it also differs from cases of overdetermination in which [*Q*] itself does not provide a full ground: [*P*], [*Q*] redundantly ground [$P\vee (P\wedge Q)$]. Unlike [*Q*], the normative law does not make any distinctive contribution.

18 See Bader (2017: §4).

19 See Bader (2017: 123–24).

20 For example, the interesting question of how different grounding relations interact with respect to, e.g., chaining principles. For a detailed discussion, see Berker (2018). In the present context, we can sidestep those issues by focussing on normative facts that are non-derivative within the normative domain.

21 For the purposes of this paper, I assume a notion of fundamentality that can be specified in terms of ground. For deviating approaches to fundamentality, see, e.g., Sider (2011).

22 See the references in fn. 20.

23 See Rosen (2017c: 155).

24 To be clear: Multiple realizability is a reason to be skeptic about reduction. However, non-naturalists, especially compared to non-reductive *naturalists* need more: They need metaphysical discontinuity.

25 An anonymous reviewer wonders whether Rosen can give us a reason: normative facts are not fully metaphysically grounded in non-normative facts and their essence does not involve non-normative facts. But the essence of normative facts involves the existential fact that there are normative laws that specify full *non-normative* n-grounds which can team up with these non-normative facts to form a full metaphysical ground. All I’m saying here is that it is not clear to me that the claim that it is metaphysically necessary that there is a non-normative complete explanation is really compatible with metaphysical discontinuity of the two fact types.

26 See Leary (2021), Bengson et al. (2024: 193). Bengson et al. (2024: 181) even endorse the claim that it is essential to moral properties that their instantiations are fully grounded in non-normative facts.

27 See Bengson et al. (2024: 73), Leary (2021: 807).

28 See, e.g., FitzPatrick (2008).

29 See, e.g., (Fine 2012: 50). On Fine’s account this is also called ‘partial strict ground’. Note that according to the definition, every full ground is a partial ground. When I speak of partial grounds, I often mean partial grounds that are not full grounds. Since in what follows we are only concerned with proper partial grounds in this sense, I will continue to drop the qualification.

30 See Leuenberger (2020), Trogdon and Witmer (2021). See also (Fine 2012: 53).

31 This and the example to follow are variants of examples discussed in Leuenberger (2020) and Trogdon and Witmer (2021).

32 For example, Trogdon and Witmer (2021) offer an analysis of full ground in terms of partial ground and the notion of nothing over and aboveness.

33 Fine (2012) invokes a notion of weak full ground according to which every truth can and indeed does ground itself. Based on that notion, Fine accounts for partial grounds that are not part of a full ground in the irreflexive sense we employed so far.

34 Thanks to an anonymous reviewer for raising this issue.

35 An anonymous reviewer wonders whether the relevant difference is that in the atom example we seem to have a necessary condition, while in the normative case the condition seems to be sufficient. Note that even if that is true, modal sufficiency does not entail explanatory sufficiency, and explanatory sufficiency is what is at issue, see in particular Sect. 5.

36 See Enoch (2011: 4, 80–81).

37 Note that Trogdon and Witmer (2021: 265) define full ground in terms of partial ground such that full ground ‘ensures that the grounded is nothing over and above the grounds’.

38 Note that UPGAp thus also satisfies a desideratum of non-naturalist approaches defended in Leary (2021: 791), according to which non-naturalism better not turn out to be trivially true, but depends on some feature of the normative itself.

39 For example, [Sam is in the office] fully grounds [Someone is in the office], but it is not maximal, given that Sarah is also in the office.

40 Berker (2019: 928, emphasizes added).

41 Leuenberger (2020: 2654).

42 There is a vast literature on normative supervenience. For an overview, see McPherson (2021).

43 See, e.g., Hattiangadi (2018), Rosen (2020).

44 For reasons discussed in the works cited in the previous footnote.

45 Thanks to an anonymous reviewer for asking me to clarify how UPGAp can deal with SC.

46 The examples presuppose a hyperintensional individuation of facts. In the present context, however, this assumption is legitimate because non-naturalists are committed to it anyway, if they are willing to accept supervenience, see Jackson (1998).

47 Another class of putative examples are facts like [*F* & the number 2 is self-identical]. Yet, if one thinks that facts like [The number 2 is self-identical] are grounded in an empty base, one might think that *F* fully grounds the conjunctive fact, see Kappes (2021).

48 See Russell and Restall (2010).

49 See, e.g., Raz (1990), Scanlon (2014), Schroeder (2007).

50 The Reasons-First approach consists of two claims: i) some reasons facts are normatively fundamental and ii) all other normative facts can ultimately be explained in terms of reasons.

51 I take this to be the standard view in the reasons debate. However, some accounts take reasons to be mental states, e.g., Davidson (2001); others allow that false propositions can be reasons, e.g., Schroeder (2007). The arguments discussed in this section apply most straightforwardly to the standard view, but matter to all views that take non-normative facts to partially ground reasons facts and that do not want to incur naturalist commitments.

52 See, for example, Berker (2019).

53 Berker (2019: 931, my emphasis).

54 I’m not convinced by Berker’s suggestion why that principle should be denied. He writes: ‘Compare: does [p] partially make it the case that [[p] is not a river] obtains? Intuitively, no.’ First, I’m not sure that I share this intuition. Second, the two cases seem to be disanalogous in a relevant sense: Facts are not rivers independently of what any particular fact is about. By contrast, no fact is a reason independently of what it is about. This suggests that facts contribute to facts about their normative role. It is also noteworthy that negative and positive facts might be grounded in very different ways. In what follows I will provide an argument for the view Berker criticizes.

55 Other partial grounds of the reasons fact that one might consider are facts about Sarah’s existence or Sarah’s ability to $\varphi$.

56 Scanlon (2014: 40).

57 Rosen (2017b: 858–859).

58 Scanlon (2014: fn. 35, p. 37, emphasis added). Fine himself mentions that, strictly speaking, we are concerned with universal claims of the form $\forall x(Fx\rightarrow Gx)$ for non-normative *F* and normative *G* (Fine 2002: 272–273).

59 Rosen (2017b: 858).

60 The importance of the latter point has been emphasized by Schroeder (2015).

61 According to Schroeder, we can approximate necessary pure normative claims by means of counterfactual claims like ‘if *p* were true, and *x* were in circumstances *c*, then one reason for *x* to do *a* would be *p*’(Schroeder 2015: 196). Schroder emphasizes that the account would not succeed, if *R*(*p*, *x*, *c*, *a*) just *meant* something counterfactual about when *p* would be a reason for *x*.
